# Draft Genome Sequences of Four Novel Strains of Microbes Isolated from *Lepidocephalichthys guntea*

**DOI:** 10.1128/MRA.00621-21

**Published:** 2021-09-23

**Authors:** Chandana Basak, Nibendu Mondal, Subhajit Sen, Jagannath Sarkar, Wriddhiman Ghosh, Ranadhir Chakraborty

**Affiliations:** a Omics Laboratory, Department of Biotechnology, University of North Bengal, Siliguri, West Bengal, India; b Department of Microbiology, Bose Institute, Kolkata, West Bengal, India; Indiana University, Bloomington

## Abstract

We report the draft genome sequences of four bacterial strains (all of which are putatively novel species) belonging to four different genera. The Gram-positive *Bacillus* sp. strain GG161 and *Rhodococcus* sp. strain GG48 and the Gram-negative *Achromobacter* sp. strain GG226 and *Shigella* sp. strain GCP5 were all isolated from the gut of the optionally intestine-breathing freshwater fish *Lepidocephalichthys guntea*.

## ANNOUNCEMENT

Fish intestines harbor complex microbial communities that play key physiological roles for the fish, including digestion, assimilation, xenobiotic compound degradation, homeostasis, and immune functions (1, 2). In this study, gut-inhabiting bacteria were isolated from the freshwater bottom-dwelling fish *Lepidocephalichthys guntea* (Hamilton, 1822) (3), which can survive with low levels of dissolved oxygen (4) due to its capacity to breathe through the intestine (5). Live fish retrieved from the Magurmari River of the North Bengal University campus (26.7095°N, 88.3542°E) were brought to the laboratory, anesthetized using clove oil (6), and dissected to remove the digestive tract under aseptic conditions. The digestive tracts were chopped into pieces and homogenized in phosphate-buffered saline (PBS) (pH 7.4). The homogenized samples were serially diluted (1:10) in PBS, pour plated in Luria agar under aerobic conditions (7), and incubated at 30°C for 72 h. In this process, four clonally purified bacterial cultures were isolated and identified as strains of *Achromobacter*, *Bacillus*, *Rhodococcus*, and *Shigella* based on their 16S rRNA gene sequence similarities (8) to validly published bacterial species. While strains of *Shigella* and *Bacillus* are often reported from fish intestines, there is no report of *Achromobacter* or *Rhodococcus* being isolated from this environment. The study was conducted according to the guidelines of the Committee for the Purpose of Control and Supervision of Experiments on Animals (CPCSEA) (9) through the Institutional Animal Ethics Committee (application 840/GO/Re/S/04/CPCSEA [application date, 27 January 2021]).

Genomic DNA of the isolates was extracted using the PureLink genomic DNA isolation kit (Thermo Fisher Scientific, USA) and quantified using a SPECTROstar Nano microplate reader (BMG Labtech, Germany) (10). Genomic DNA was enzymatically sheared, and a library was constructed using the Ion Xpress Plus fragment library kit (Thermo Fisher Scientific). Approximately 330-bp DNA sequences were selected using E-Gel SizeSelect 2% agarose gels (Thermo Fisher Scientific) and sequenced on an Ion 540 chip with the Ion S5 system (Thermo Fisher Scientific). Before sequence retrieval from the Ion S5 system, low-quality (scores of >Q20) and polyclonal reads were filtered out and the adaptor sequences were trimmed using the inbuilt PGM software. The high-quality reads were assembled into contigs using SPAdes v3.13.0 with default parameters (11). The draft genome sequences were annotated using the NCBI Prokaryotic Genome Annotation Pipeline (PGAP) and Rapid Annotations using Subsystems Technology (RAST) software. The analyses of the genomes are summarized in Table 1.

**TABLE 1:** Summary information on the genomes of the four new bacterial strains isolated from the intestine of *Lepidocephalichthys guntea*

Characteristic	Data for:			
	*Achromobacter* sp.	*Rhodococcus* sp.	*Shigella* sp.	*Bacillus* sp.
Strain name	GG226	GG48	GCP5	GG161
BioProject accession no.	PRJNA668892	PRJNA669658	PRJNA669212	PRJNA669660
SRA accession no.	SRR14817175	SRR14820309	SRR14813644	SRR14820274
BioSample accession no.	SAMN16427092	SAMN16451880	SAMN16456367	SAMN16451878
GenBank accession no.	JADCNH000000000	JADDIS000000000	JADDOI000000000	JADDIT000000000
No. of high-quality reads	8,455,791	11,334,927	11,221,961	11,603,137
Mean read length (bp)	195	176	203	189
No. of contigs	118	107	89	47
Genome size (bp)	4,716,310	5,164,828	4,908,902	3,715,557
Genome coverage (×)	266	183	379	747
GC content (%)	67.0	68.8	50.7	41.3
*N*_50_ (bp)	102,141	110,296	213,545	371,380
*L*_50_	14	14	8	3
Total no. of genes	4,477	4,881	4,662	3,857
No. of coding genes	4,187	4,619	4,380	3,682
No. of rRNAs	7	5	15	15
No. of tRNAs	47	52	74	76
No. of noncoding RNAs	4	3	7	5
No. of pseudogenes	232	202	186	79
No. of CRISPR arrays	2	0	2	0

Searches against the CARD v3.1.1 database (12) showed that only the genome of *Shigella* sp. strain GCP5 included genes for resistance against fluoroquinolones, macrolides, cephalosporins, cephamycins, penems, tetracycline, aminoglycosides, carbapenems, glycylcyclines, peptide antibiotics, aminocoumarins, rifamycins, phenicols, triclosan, monobactams, benzalkonium chloride, and rhodamine. As revealed by RAST (13), all four strains possessed genes conferring resistance to copper, cobalt-zinc-cadmium, and selenium, as well as those involved in degrading xenobiotic compounds such as toluene and xylene. *Shigella* sp. strain GCP5 and *Achromobacter* sp. strain GG226 also carried genes for formaldehyde detoxification.

### Data availability.

The whole-genome shotgun projects have been deposited in DDBJ/ENA/GenBank, while the raw sequence reads have been deposited in the NCBI Sequence Read Archive (SRA). All relevant accession numbers are given in Table 1.
